# Syringomyelia.—I

**Published:** 1891-06-13

**Authors:** 


### THE PRACTITIONER'S MIRROR AND RETROSPECT.

[*The Editor will be glad to receive offers of co-operation and contributions from members of the profession. There is no doubt that much valuable material full of interest and instruction is at present lost to science. This is largely so in the case of* (1) *the younger and less-known members of the hospital staff* (2) *those connected with cottage hospitals, and* (3) *the great body of medical practitioners not attached to any institution. The co-operation of all is cordially invited to enable the Editor to make* THE HOSPITAL *of the utmost possible use and value to the profession. Specialists willing to review books on their special subjects are invited to communicate this fact at once. All letters should be addressed to* THE EDITOR, THE LODGE, PORCHESTER SQUARE, LONDON, W.]

### MEDICINE.

### SYRINGOMYELIA.—I.

Syringomyelia is the formation of a cavity in the spinal cord elsewhere than in the central canal. The following is taken from an article by M. Paul Bloc in a recent number of "Brain."* Clinically, the disease is characterised by certain alterations in sensibility in association with trophic disorders, and bearing some resemblance to progressive muscular atrophy, with which it has been confounded until comparatively recently. The word syringomyelia was used for the first time by Olliviers (d'Angers), συριγγωδης, hollowed out in the shape of a pipe; μυελός, marrow. This author did not admit the existence of the central canal in the spinal cord, and he considered that the different facts brought forward in support of its existence by Brüner, Santorini, and Rachetti were to be attributed to an arrest of development. Ollivier then, while inventing the name syringomyelia, had no idea of the true nature of the disease, and of which he makes no mention. When subsequent anatomical researches had incontestably proved the normal existence of the central canal in the cord, syringomyelia was soon forgotten. It was not long, however, before observations of the abnormal dilation of this canal were made, and the majority of them were at the time looked upon as arrests of development, and the name hydromyelia was commonly given to them.

In 1870 M. Hallopeau believed that cases such as had been described by Gull, Mayer, and Schüppel ought no longer to be classed under the heading hydromyelia, but that they should be described under the name of "Diffuse Periependymal sclerosis." This form of myelitis was, in fact, characterised by the presence of groups of separate cavities in the spinal cord, due to an inflammatory sclerosis of the neuroglia, of which these cavities constitute one method of development. Soon after this Charcot and Joffroy suggested that the formation of these cavities was due to a granular disintegration (the granular disintegration of Clarke). This affection did not really take a definite place in the description of diseases until the works of Schültze, Professor of Derpat, and Kahler of Prague, who have established—as M. Charcot proclaimed in one of his lectures—that a certain number of particular symptoms might be traced to that organic lesion, and might be permitted even to determine the principal peculiarities. It is, however, necessary to notice that M. Charcot, as early as 1874, had pointed to syringomyelia as one of the possible causes of muscular atrophy of spinal origin. Syringomyelia comprises, etymologically speaking at least, the study of all the affections in which cavities in the spinal cord are to be observed. We have, however, seen that cases in which cavities occur in the spinal cord are to be excepted, and are included in the term hydromyelia.

It remains then to consider syringomyelia: (1) As the result of myelitis, and (2) as the result of glioma. The lesion is, as a rule, bilateral. The cavities vary in size from a mere slit to such as occupy the whole extent of the cord, of which only sufficient remains to form the thin wall of the cavity. The extent of the cavity may likewise vary in longitudinal direction. It may occupy the whole length of the cord (Schültze), and may encroach upon the medulla. The ascending root of the trigeminal, the olivary body, the nucleus of the hypoglossal may be invaded. It may also extend as low as the *filum terminale*. The cervical enlargement is most frequently affected. The central canal is usually unaffected; occasionally it communicates to a certain extent with the pathological cavity. The microscopical changes consist, according to the majority of authors, in a neoplastic hyperplasia of the neuroglia of the grey or gliomatous matter; according to other authorities, in an inflammatory hyperplasia or myelitis. One can conceive that the parts of the spinal cord adjacent to the gliomatous hyperplasia are more or less affected by it. They are either compressed as the lesion extends, or separated out by an infiltration of the neuroglia, and are consequently displaced or undergo alterations. In the latter case hæmorrhage, inflammation, and degeneration may occur.

As regards the peripheral nerves, Holschewnikoff was the first to show in a case of syringomyelia that the posterior roots of the cervical nerves lost their myeline and would not stain by Weigert's method. M. Dejerine has shown that the intramuscular nerves of the unaffected muscles are normal, while the intramuscular nerves of the muscles that were atrophied were very much altered, and were only represented by the empty sheaths.

(*To be continued.*)

* "Brain," pt. li.

